# Sex- and age-specific musculoskeletal adverse events of isotretinoin: disproportionality analysis of the FAERS database combined with a clinical case series

**DOI:** 10.3389/fphar.2026.1904582

**Published:** 2026-09-11

**Authors:** Yidan Mu, Gang Xue, Wenjing Li, Guangping Li, Xiaoyue Du, Desheng Zheng, Man Jiang

**Affiliations:** 1 School of Pharmacy, Qingdao University, Qingdao, China; 2 University of Electronic Science and Technology of China, Chengdu, China; 3 Department of Pharmacy, The Affiliated Hospital of Qingdao University, Qingdao, China; 4 School of Computer Science and Software Engineering, Southwest Petroleum University, Chengdu, China

**Keywords:** adverse event, disproportionality analysis, isotretinoin, musculoskeletal, stratified analysis

## Abstract

**Background:**

Isotretinoin is widely used for the treatment of acne and has been associated with musculoskeletal and connective tissue disorder adverse events However, existing research remains limited in terms of large-scale systematic investigations of these adverse effects. This study utilized the FDA Adverse Event Reporting System the Canadian Vigilance Adverse Reaction Online Database (CVARD), and clinical data to comprehensively characterize the potential musculoskeletal and connective tissue toxicity of isotretinoin and to identify population-specific differences in adverse event patterns.

**Methods:**

Disproportionality analysis was conducted using the reporting odds ratio (ROR) method to identify potential safety signals. Subgroup analyses were performed according to sex, age, and dosage. Stratified ROR analyses were further conducted to evaluate reporting patterns associated with the concomitant use of oral contraceptives, tetracyclines, and prednisone. Time-to-onset characteristics were assessed using Weibull distribution analysis and cumulative temporal reporting distribution curves. Supplementary analyses were performed using external data from the CVARD database and clinical case series from the Affiliated Hospital of Qingdao University.

**Results:**

A total of 30 positive safety signals were identified. The strongest signals were observed for epiphyses premature fusion (ROR = 216.92), SAPHO syndrome (ROR = 76.41), epiphyseal disorder (ROR = 51.42) and sacroiliitis (ROR = 45.36). Stratified analyses demonstrated male-predominant signals for SAPHO syndrome, epiphyses premature fusion, sacroiliitis, arthritis, and arthralgia (ROR ratios: 1.42–7.85). Adolescents exhibited significantly higher ROR ratios than adults for tendonitis, intervertebral disc degeneration, costochondritis, back pain, and arthralgia (ROR ratios: 2.30–7.41). Epiphyses premature fusion was predominantly reported in adolescents (n = 66, vs 2 adults), but the ROR for the adolescent versus adult comparison was not estimable owing to sparse data. All groups belonged to the early-failure pattern.

**Conclusion:**

Sex- and age-stratified analyses identified several exploratory differences in reporting patterns. These exploratory findings provide hypothesis generating insights that warrant further validation in prospective clinical studies.

## 1 Introduction

Acne is a chronic inflammatory skin disease involving the pilosebaceous unit, with a complex pathogenesis including excessive sebum secretion, abnormal follicular keratinization, proliferation of Cutibacterium acnes, and inflammatory responses (Dréno, 2017). As a natural derivative of vitamin A, isotretinoin (13-cis-retinoic acid) has been widely used for the treatment of severe acne since its approval by the U.S. Food and Drug Administration (FDA) in 1982 (Villani et al., 2022). However, the widespread use of isotretinoin has raised concerns regarding its multisystem adverse effects and potential toxicities.

At present, well-established screening and monitoring strategies are recommended in clinical guidelines for the severe teratogenicity, hepatotoxicity, and dyslipidemia associated with isotretinoin (Reynolds et al., 2024). Previous studies have also systematically evaluated its psychiatric AEs risks (Nie et al., 2025), reproductive toxicity (Altınöz Güney and Koç, 2026), and ocular toxicity (Tao et al., 2025). Although musculoskeletal and connective tissue disorder AEs associated with isotretinoin are commonly reported in clinical practice, ranging from common symptoms such as myalgia, arthralgia, and back pain to more serious conditions including sacroiliitis and epiphyses premature fusion (Almutairi et al., 2024; Ruchitha et al., 2025), comprehensive evaluations of these adverse effects remain limited.

A disproportionality analysis comparing isotretinoin with topical retinoids revealed stronger reporting signals for musculoskeletal disorders among oral isotretinoin (Szilagyi et al., 2026). Previous studies have suggested an association between isotretinoin use and sacroiliitis, with lower back pain potentially serving as a clinical manifestation of this condition (Zhao et al., 2025; Karaosmanoğl et al., 2020). Regarding skeletal development and bone metabolism, available evidence has not consistently demonstrated reductions in bone mineral density associated with isotretinoin, although a mild reduction in femoral neck bone mineral density cannot be excluded (Ershadi et al., 2025). In pediatric and adolescent populations, isotretinoin use has been associated with epiphyses premature fusion (Alazawi and Hendriksz, 2021). Furthermore, reports of elevated creatine kinase levels suggest the potential isotretinoin-induced skeletal muscle injury (Mülkoğl et al., 2021).

Therefore, this study analyzed AE reports from the FAERS database (U.S. Food and Drug Administration, 2026) between the first quarter of 2004 and the fourth quarter of 2025. Disproportionality analyses were conducted to systematically characterize musculoskeletal and connective tissue disorder AEs associated with isotretinoin and to identify sex- and age-specific reporting patterns. Supplementary external contextualization was subsequently performed using the CVARD (Health Canada Canada Vigilance Adverse, 2026), together with the clinical case series from the Affiliated Hospital of Qingdao University. By integrating pharmacovigilance and real-world clinical data, this study aimed to provide a comprehensive assessment of isotretinoin-related musculoskeletal and connective tissue safety signals and their potential clinical implications.

## 2 Materials and methods

### 2.1 Data sources and process

A retrospective pharmacovigilance study was performed using data from the FAERS. Reports involving isotretinoin submitted between the first quarter of 2004 and the fourth quarter of 2025 were extracted. The downloaded data included patient demographic information, drug information, indication information, patient outcomes, AE information, reporting source information and the start and end dates of drug treatment. Records were integrated using the unique identifier “PRIMARYID,” and duplicate reports were removed in accordance with the FDA’s official data cleaning guidelines. Records were sorted by CASEID, FDA_DT and PRIMARYID. For records sharing identical CASEID, only the report with the maximum FDA_DT value was retained. If both CASEID and FDA_DT were identical across entries, the record with the larger PRIMARYID was kept. Data were processed using MySQL 8.0 and Python 3.10, utilizing the pandas (v1.5.3) and NumPy (v1.24.1) packages. GraphPad Prism (v10.6.0) was used for data visualization.

**FIGURE 1:**
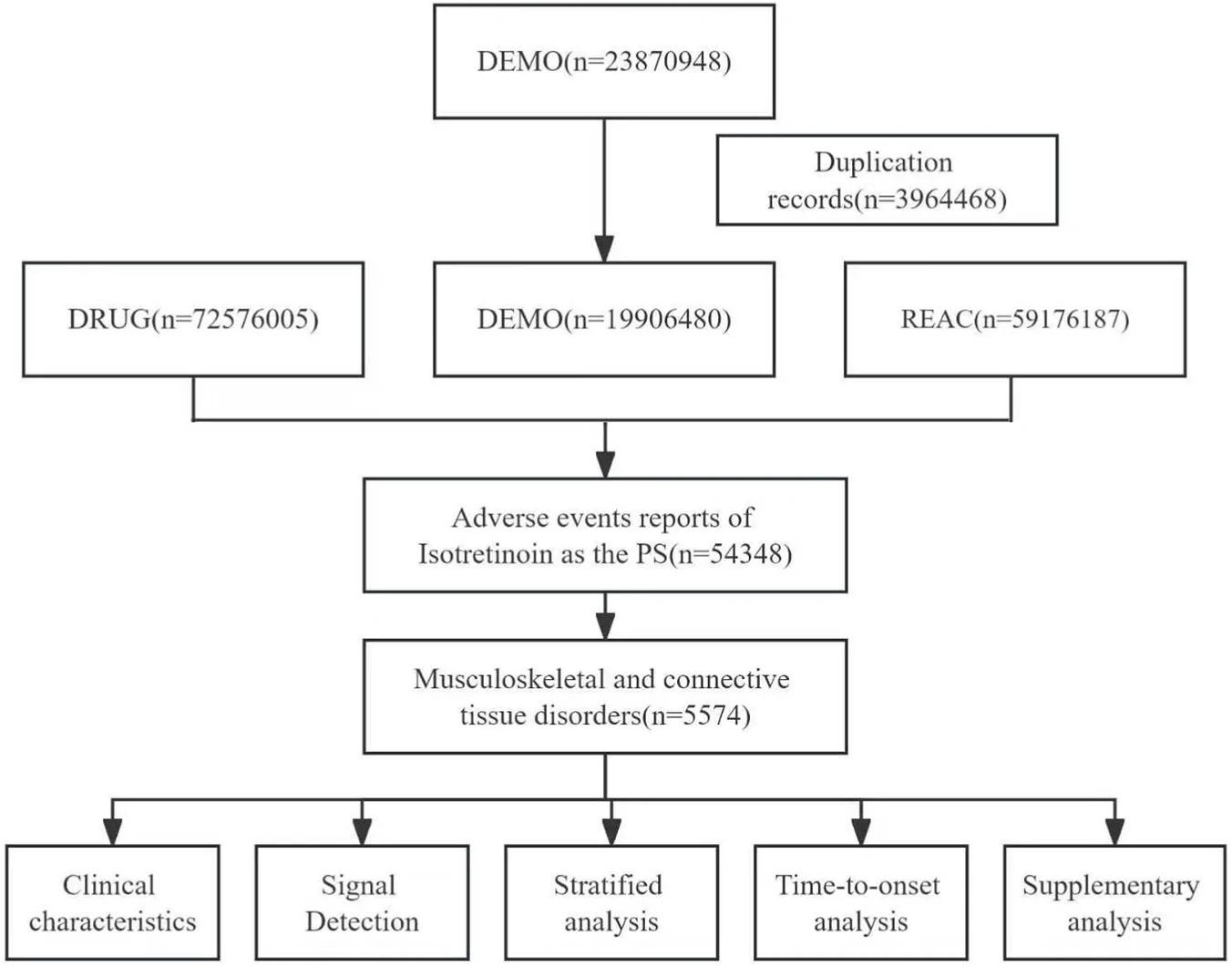
Workflow for selecting musculoskeletal and connective tissue disorders adverse event reports among isotretinoin users from the FAERS database.

The drug information dataset was searched using both generic and brand names of isotretinoin, including “isotretinoin”, “Absorica”, “accutane”, “Amnesteem”, “Claravis”, “Clarus”, “Epuris”, “Myorisan”, “Sotret” and “Zenatane”. Only AE reports in which isotretinoin was identified as the primary suspect (PS) drug were included. AEs were coded and standardized using preferred terms (PTs) according to the Medical Dictionary for Regulatory Activities (MedDRA) version 28.1, and further categorized by system organ class (SOC). Reports classified under the SOC of musculoskeletal and connective tissue disorders (SOC code: 10028395) were selected for analysis. Figure 1 shows the selection flowchart for musculoskeletal and connective tissue disorder AE reports among isotretinoin users from FAERS. We descriptively summarized demographic and clinical characteristics, including sex, age, weight, reporter type, outcome, indication, and reported country, as frequencies and percentages. For reports with multiple serious outcomes, we counted each report only once and prioritized the most severe event according to the following hierarchy: Death > Life-Threatening > Hospitalization > Disability > Required Intervention > Congenital Anomaly > Other Serious.

### 2.2 Signal detection

Disproportionality analysis using the reporting odds ratio (ROR) method was employed to detect potential safety signal (Fusaroli et al., 2024), with the formula shown in Table 1. The basic unit for disproportionality analysis was drug-event pairs derived from unique individual safety reports. For each eligible report, we extracted the suspect drug (isotretinoin) and all associated AE to form separate drug-event pairs for 2 × 2 contingency table construction and ROR estimation. The reference group consisted of all FAERS reports involving drugs other than isotretinoin. A positive signal was defined as a target AE count of ≥5 with the lower limit of the 95% confidence interval (CI) > 1, indicating a potential disproportionate reporting signal for isotretinoin compared with all other drugs in the database. Higher ROR values indicate stronger reporting associations but do not imply established causality.

**TABLE 1:** Calculation of Reporting Odds Ratio (ROR).

Drugs	Target AE	Other AE	Total
Target drugs	a	b	a + b
Other drugs	c	d	c + d
Total	a + c	b + d	a + b + c + d

The calculation formulas are shown below.

ROR = (a/c)/(b/d) = ad/(bc); 95%CI = exp[ln(ROR)±1.96*sqrt(1/a + 1/b + 1/c + 1/d)]

### 2.3 Stratified analysis

To explore potential co-occurrence patterns among reported events, we selected PTs with positive ROR signals and more than 10 reports for Spearman correlation analysis. A correlation matrix was constructed based on the presence of each PT within individual reports, to assess pairwise co-occurrence correlations across distinct reports.

To explore the influence of potential confounding factors and identify susceptible populations, stratified disproportionality analyses were conducted. At the SOC level, the dataset was stratified by demographic and clinical variables, including sex (male vs. female), age group (adolescents [12–17 years] vs. adults [18–44 years]), indication (acne-related vs. unknown), active comparator (tretinoin, tazarotene), and reporter type (healthcare professionals, lawyers, consumers). Subsequently, additional PT-level stratified analyses of the 30 positive signal PTs were undertaken to investigate sex- and age-related disparities in reporting signals. Subgroup differences (male vs. female, adolescents vs. adults) were evaluated by calculating ROR ratios. Statistical significance was determined via a *Z*-test based on the logarithmic transformation of RORs and their standard errors (*P* < 0.05, two-tailed).

Daily dosage (mg/kg/day) was derived from the drug information dataset. For reports with a direct mg/kg/day dosage value recorded in the “DOSE_VBM” field, this value was used as the daily dosage. For reports where only the per-administration dose and dosing frequency (BID, QD, TID) were available, the total daily dose (mg/day) was calculated by multiplying the per-administration dose by the number of administrations per day, and then divided by the patient’s body weight (kg) to obtain the weight-normalized daily dose (mg/kg/day). Reports with missing dose or body weight information were excluded. The resulting daily dosages were categorized into three groups: <0.5, 0.5≤ and ≤1, and >1 mg/kg/day. For each PT, the number of reports falling into each dosage group was tabulated to describe the distribution of AEs across different isotretinoin dose levels.

We calculated the frequency of concomitant medications among isotretinoin users with musculoskeletal and connective tissue disorder AEs. ROR analyses were further performed for oral contraceptives (the most frequently reported), tetracyclines, and prednisone, given their known potential to induce musculoskeletal AEs.

### 2.4 Time-to-onset analysis

The time-to-onset of isotretinoin-related musculoskeletal and connective tissue disorder AEs was calculated as the interval between the therapy start date (START_DT) and the event onset date (EVENT_DT) in FAERS database. We excluded reports with missing date information, implausible records in which EVENT_DT preceded START_DT, and records with a time-to-onset value exceeding 730 days.

Cumulative temporal reporting distribution curves for AEs related to isotretinoin musculoskeletal and connective tissue disorder AEs were constructed, and the Log-rank test was used to compare differences in the time-to-onset between different groups. In this study, Weibull distribution analysis was used to characterize the temporal patterns of AE occurrence, with subgroup analyses performed according to sex and age.

### 2.5 Supplementary analysis

To contextualize the FAERS findings with additional real-world data, we conducted a supplementary analysis using the CVARD and clinical case series. CVARD data from 1965 to 2025 were downloaded. Duplicate records were removed based on “REPORT_ID”. The “Reactions”, “Report_Drug”, and “Reports” data were linked by “REPORT_ID”. Reports in which isotretinoin was listed as the suspected drug were included in the analysis. AEs were harmonized to PTs using MedDRA version 28.1. Signal detection was performed as described in Section 2.2.

To clinically contextualize pharmacovigilance signals derived from the FAERS database, we performed a retrospective medical chart review enrolling 1,322 patients receiving isotretinoin treatment at The Affiliated Hospital of Qingdao University from 1 January 2025 to 31 December 2025. Subsequent screening for isotretinoin-related musculoskeletal AE cases was conducted based on inclusion/exclusion criteria and causality evaluation. The inclusion criteria were as follows: (1) patient age ≥12 years old; (2) confirmed clinical diagnosis of acne; (3) new-onset musculoskeletal symptoms that developed during isotretinoin administration or within 30 days following drug discontinuation. Patients were excluded if they had pre-existing rheumatoid arthritis or ankylosing spondylitis, to minimize confounding. All candidate cases were independently adjudicated by two clinical pharmacists. Any discrepancies between the two reviewers were resolved via mutual consensus or arbitration by a third senior investigator. The causal relationship between isotretinoin exposure and reported musculoskeletal AEs was evaluated using the World Health Organization-Uppsala Monitoring Centre (WHO-UMC) causality assessment system, which stratifies causal associations into six tiers: certain, probable/likely, possible, unlikely, conditional/unclassified, and unassessable. For the current clinical cohort, only cases rated as certain, probable, or possible were retained to guarantee conservative, clinically relevant evaluation. The detailed patient-screening procedure is presented in Supplementary Figure S2. All included AE terms were mapped to the PT level of MedDRA to ensure terminological consistency with the primary FAERS database analysis.

## 3 Results

### 3.1 Descriptive analysis

This study identified 5,574 reports of isotretinoin-related musculoskeletal and connective tissue disorder AEs in the FAERS database from the first quarter of 2004 to the fourth quarter of 2025. Females constituted 50.2% of the cohort, and males 40.5%. In age stratification, the 12–17 group (24.3%) and the 18–44 years group (34.4%) showed higher proportions. Reports were most common in individuals weighing 50–100 kg (35.3%). Physicians were the most frequent reporters (31.0%), followed by consumers (26.7%) and lawyers (19.8%). Regarding clinical outcomes, the three most frequently reported outcomes were “Other Serious” (33.0%), hospitalization (12.0%), and disability (6.4%). The three most frequent indications were acne (48.2%), product used for unknown indication (23.1%), and cystic acne (8.7%). Regarding geographic distribution, the United States accounted for 80.8% of reports, followed by the United Kingdom (2.4%) and Turkey (2.3%). Detailed demographic information is presented in Table 2, and the annual reporting distribution from 2004 to 2025 is illustrated in Supplementary Figure S1.

**TABLE 2:** Clinical characteristics of patients reporting adverse events of musculoskeletal and connective tissue disorders.

Characteristic	Number of reports (n)	Proportion (%)
Total number of reports	5,574	​
Sex		
Female	2,796	50.2%
Male	2,257	40.5%
Missing	521	9.3%
Age (year)		
<12	54	0.9%
12≤and≤17	1,358	24.3%
18≤and≤44	1916	34.4%
45≤and≤59	208	3.7%
>59	45	0.8%
Missing	1993	35.8%
Weight (kg)		
<50	193	3.4%
50≤and≤100	1967	35.3%
>100	131	2.4%
Missing	3,283	58.9%
Reporters		
Physician (MD)	1730	31.0%
Consumer (CN)	1,487	26.7%
Lawyer (LW)	1,105	19.8%
Other health-professional (OT)	625	11.2%
Health professional (HP)	372	6.7%
Pharmacist (PH)	64	1.1%
Missing	191	3.4%
Outcome		
Other serious (OT)	1837	33.0%
Hospitalization (HO)	671	12.0%
Disability (DS)	356	6.4%
Required intervention to prevent permanent impairment/Damage (RI)	59	1.1%
Life-threatening (LT)	55	1.0%
Death (DE)	32	0.6%
Congenital anomaly (CA)	28	0.5%
Missing	2,536	45.5%
Indications (top three)		
Acne	2,684	48.2%
Product used for unknown indication	1,290	23.1%
Acne cystic	485	8.7%
Reported country (top three)		
United States of America (US)	4,503	80.8%
United Kingdom of great britain and northern Ireland (GB)	134	2.4%
The republic of Turkey (TR)	124	2.3%

### 3.2 Signal detection

**FIGURE 2:**
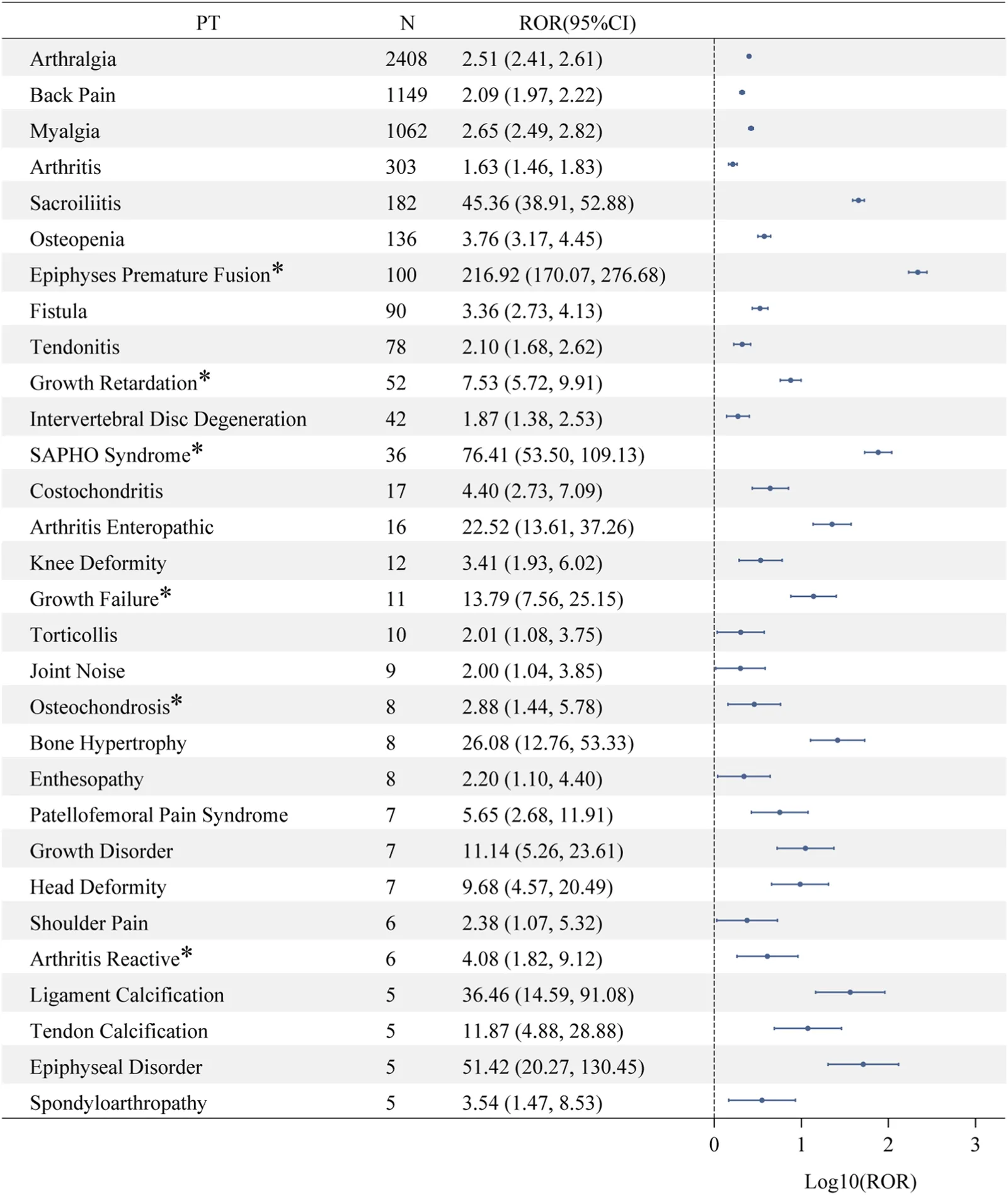
Reporting odds ratios (ROR) with 95% confidence intervals (CI) for positive signals of adverse events of musculoskeletal and connective tissue disorders among isotretinoin users in FAERS database. Note: CI, confidence interval; ROR, reporting odds ratio; N, number of drug-event pairs with suspected adverse events; PT, preferred term. *, PT classified as important medical events (IMEs).

A total of 30 positive signals corresponding to musculoskeletal and connective tissue disorder PTs were identified in the FAERS database. The five most frequently reported PTs were arthralgia (*N* = 2,408), back pain (*N* = 1,149), myalgia (*N* = 1,062), arthritis (*N* = 303), and sacroiliitis (*N* = 182). The five PTs with the highest ROR values were epiphyses premature fusion (ROR = 216.92), SAPHO syndrome (ROR = 76.41), epiphyseal disorder (ROR = 51.42), sacroiliitis (ROR = 45.36), and ligament calcification (ROR = 36.46). According to the Important Medical Event (IME) list, six of the 30 positive signals were classified as IMEs: epiphyses premature fusion, SAPHO syndrome, growth retardation (ROR = 7.53), growth failure (ROR = 13.79), osteochondrosis (ROR = 2.88), and arthritis reactive (ROR = 4.08). Further details are provided in Figure 2.

### 3.3 Stratified analysis

**FIGURE 3:**
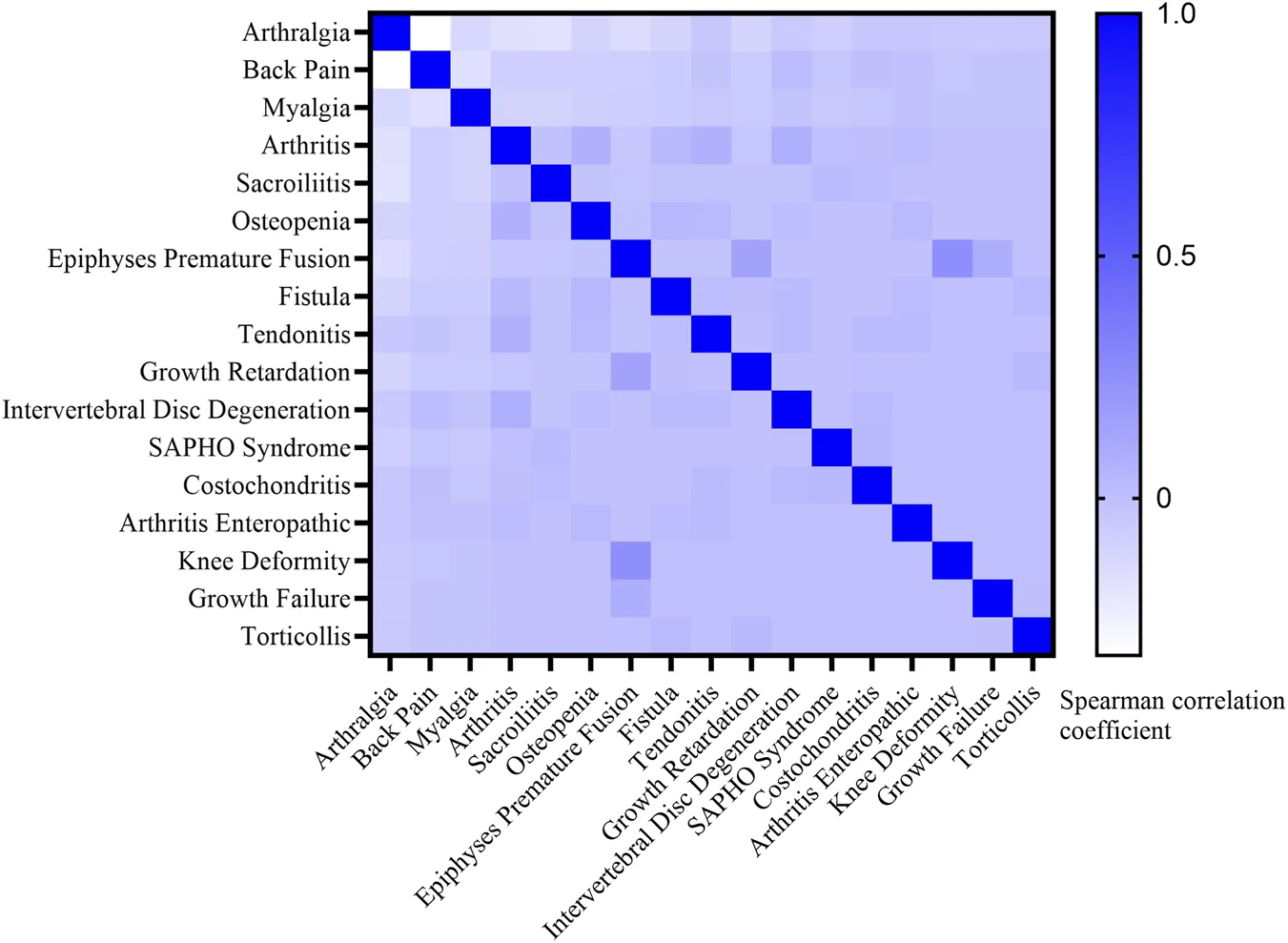
Correlation analysis of preferred terms (PTs) of musculoskeletal and connective tissue disorders with positive reporting odds ratios (ROR) signals and more than ten reports among isotretinoin users. Note: The x- and y-axes both denote the 17 eligible PTs meeting the criteria of positive ROR signals and >10 reports. Color intensity represents the pairwise Spearman correlation coefficient, where darker blue corresponds to higher correlation and lighter shades to lower correlation.

We selected PTs with positive ROR signals and more than 10 reports. Spearman correlation analysis was performed to evaluate pairwise correlations among these PTs (Figure 3). Epiphyses premature fusion was weakly positively correlated with knee deformity (*r* = 0.255, *P* < 0.001).

**FIGURE 4:**
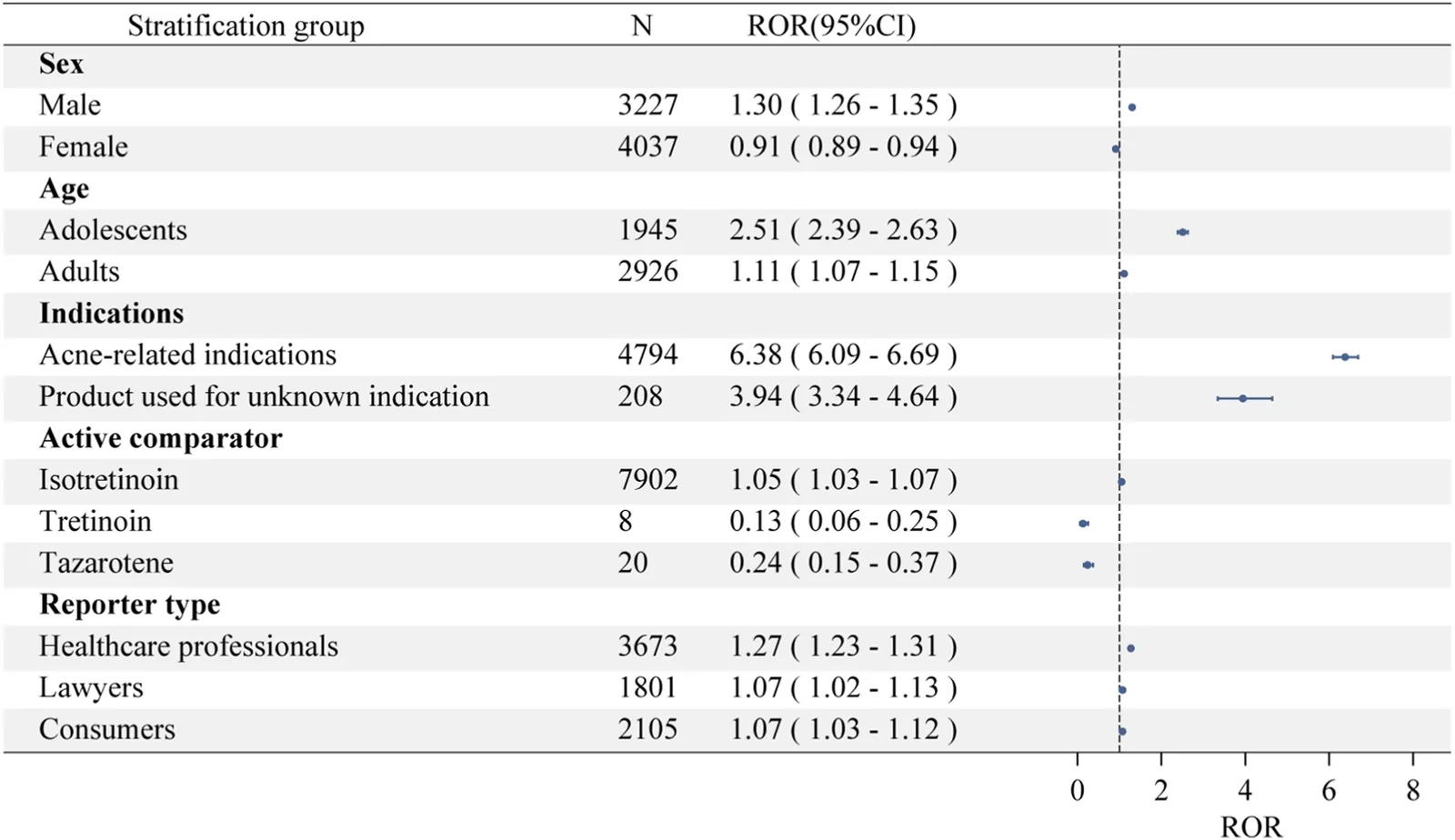
SOC-level stratified analyses of disproportionate reporting signals for isotretinoin-related musculoskeletal and connective tissue disorders. Note: SOC, system organ class; N, number of drug-event pairs with suspected musculoskeletal and connective tissue disorders in each stratum.

To assess the influence of demographic and clinical variables, stratified analyses at the SOC level were performed for isotretinoin related musculoskeletal and connective tissue AEs according to sex, age group, indication, active comparator, and reporter type (Figure 4). Notably, distinct reporting patterns emerged across demographics: males exhibited a significant positive signal (ROR = 1.30, 95% CI: 1.26–1.35), whereas females did not (ROR = 0.91, 95% CI: 0.89–0.94). Regarding age, adolescents demonstrated a stronger signal (ROR = 2.51, 95% CI: 2.39–2.63) compared to adults (ROR = 1.11, 95% CI: 1.07–1.15). The strongest overall association was observed for acne-related indications (ROR = 6.38). Furthermore, isotretinoin maintained a positive signal in the FAERS, whereas no such signals were observed for the topical retinoids (tretinoin and tazarotene) in their respective analyses. Stratified analyses by reporter type showed positive RORs for each subgroup, including healthcare professionals (ROR = 1.27), lawyers, and consumers.

**FIGURE 5:**
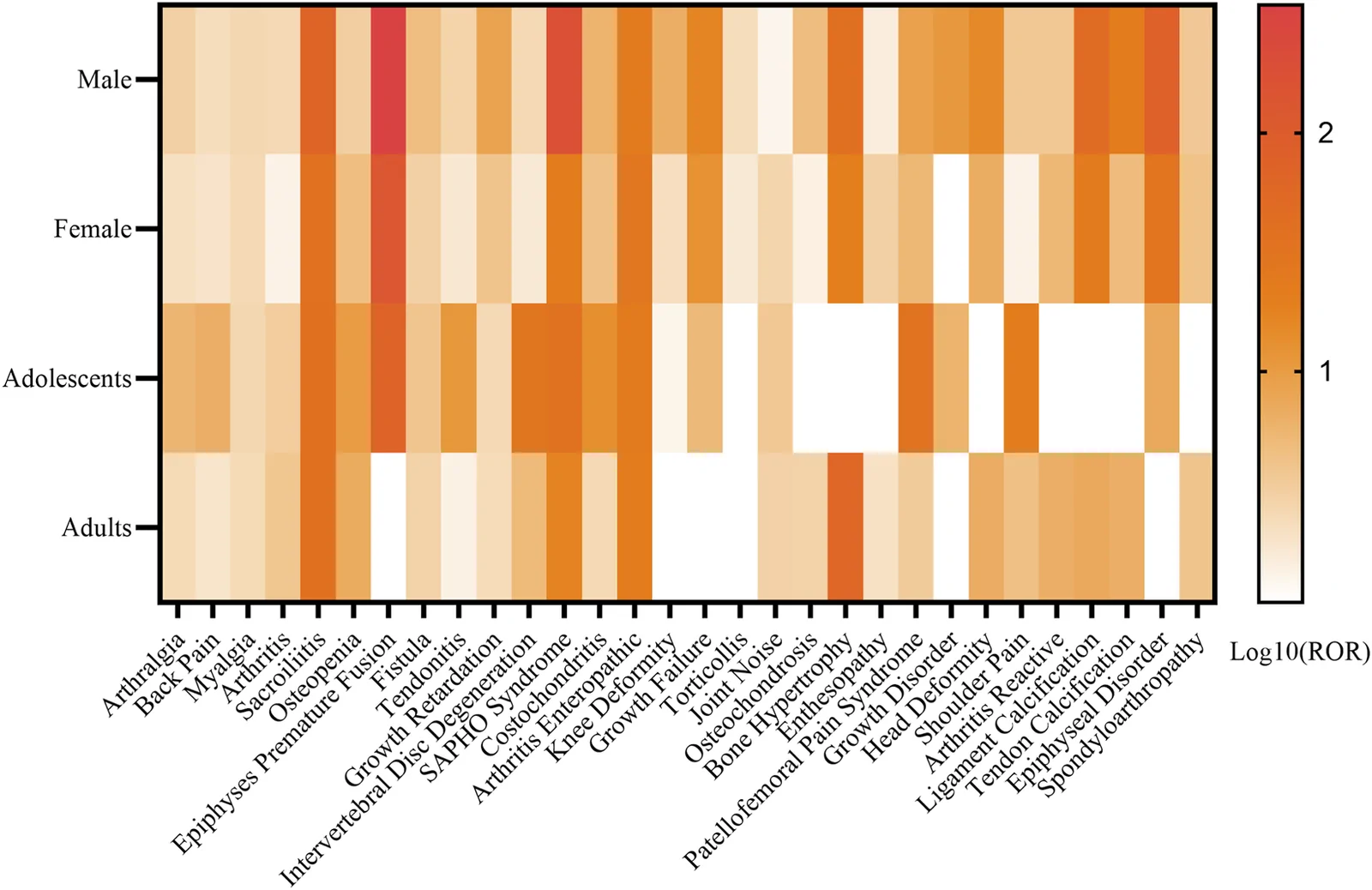
Reporting odds ratio (ROR) of 30 preferred terms (PTs) with positive disproportionality signals across the overall sample and different strata. Note: The x-axis represents 30 PTs with positive ROR signals across the overall sample. The y-axis represents the overall sample and stratifications based on the following conditions: sex (male and female) and age (adolescents and adults). Colored blocks represent the log_10_ (ROR) values. Darker red denotes higher values, while lighter shades indicate lower values.

To further characterize these isotretinoin-related safety signals, we conducted additional stratified analyses by sex and age group at the PT level for the 30 positive PTs (Figure 5). Baseline characteristics for sex- and age-stratified subgroups are compared in Supplementary Table S1 and Supplementary Table S2. Corresponding ROR ratios with 95% CIs are shown in Supplementary Table S3. For sex-stratified analysis, the ROR ratios (male vs. female) with 95% CIs indicated statistically significant male-predominant signals for arthralgia (ratio = 1.42, 95% *CI*: 1.30–1.54), arthritis (1.77, 95% *CI*: 1.37–2.29), sacroiliitis (1.89, 95% *CI*: 1.41–2.53), epiphyses premature fusion (2.72, 95% *CI*: 1.47–5.04), and SAPHO syndrome (7.85, 95% *CI*: 2.89–21.33). Osteopenia showed a lower ROR ratio in males than in females (ROR ratio: 0.70, 95% *CI*: 0.50–0.97); however, this difference did not remain statistically significant after Benjamini–Hochberg FDR correction. No statistically significant sex differences were observed for other AEs. In the age-stratified analysis comparing adolescents with adults, significantly higher ROR ratios were observed in the adolescent group for arthralgia (2.30, 95% *CI*: 2.02–2.62), back pain (3.30, 95% *CI*: 2.83–3.85), tendonitis (7.41, 95% *CI*: 4.08–13.45), intervertebral disc degeneration (5.57, 95% *CI*: 2.05–15.13), and costochondritis (5.44, 95% *CI*: 1.83–16.18). No other significant age-related differences were detected for the remaining AEs. Regarding epiphyses premature fusion, 66 reports were identified in adolescents and 2 in adults; however, because the adult subgroup contained fewer than three reports, the subgroup ROR ratio could not be reliably estimated due to sparse data, and this comparison is therefore reported as not estimable.

To describe the distribution of isotretinoin-related musculoskeletal and connective tissue disorder AEs across different dose levels, reports were stratified into three dosage groups: <0.5 mg/kg/day (*N* = 199), 0.5–1 mg/kg/day (*N* = 361), and >1 mg/kg/day (*N* = 106). A total of 4,908 reports (88.05%) were excluded from the dose-stratified analysis due to missing or uninterpretable dose entries, missing dosing frequency, or missing body weight, leaving 666 reports for the final analysis. The five most frequent preferred terms in each group are shown in Table 3. Arthralgia was the most frequently reported preferred term across all dosage groups. Myalgia and back pain were also consistently among the top five preferred terms in each group. Comparable reporting proportions of selected PTs across isotretinoin dose groups are provided in Supplementary Table S4.

**FIGURE 6:**
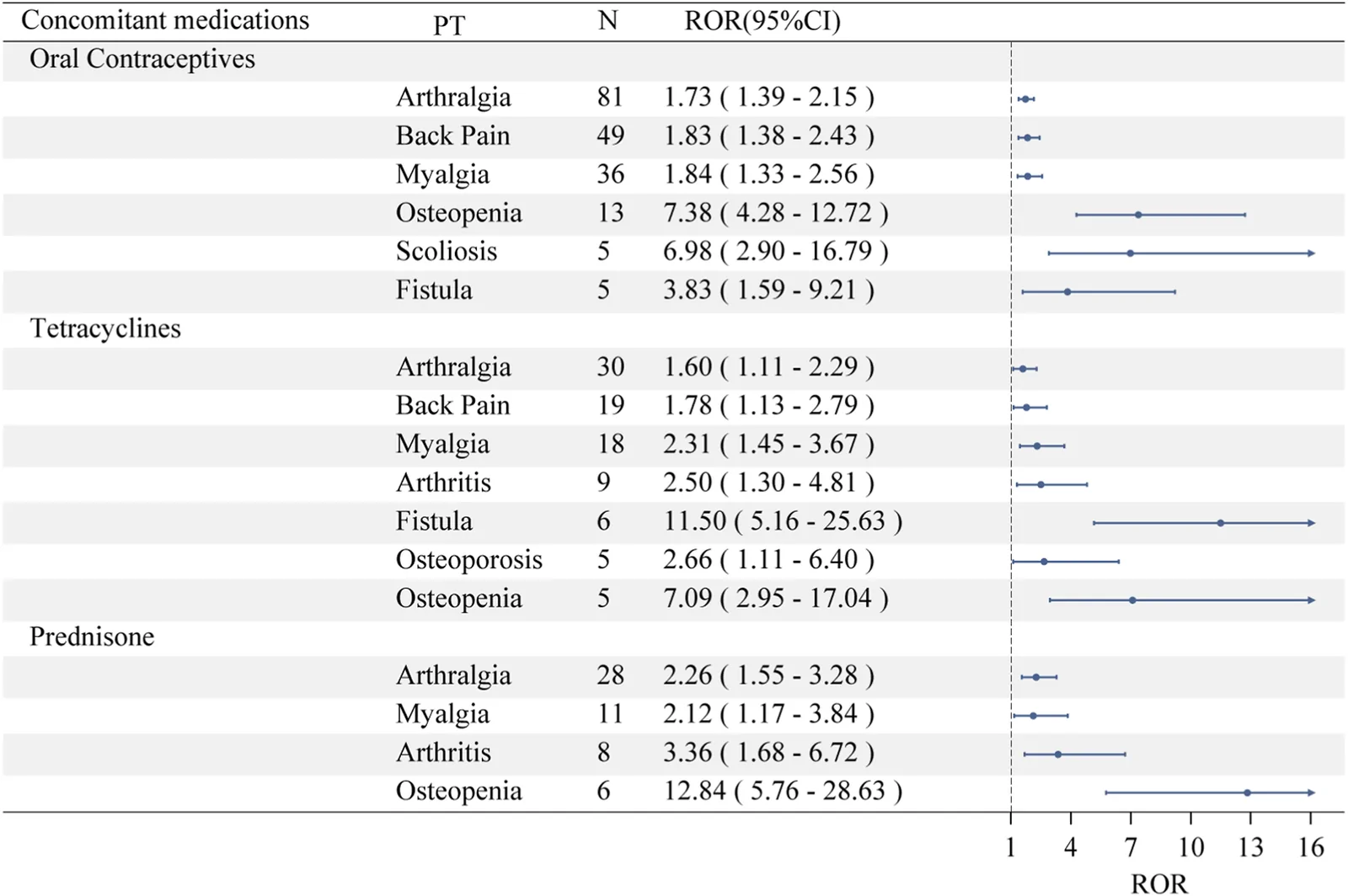
Reporting odds ratios (ROR) with 95% confidence intervals (CI) for positive signals of adverse events of musculoskeletal and connective tissue disorders associated with isotretinoin concomitant medications. Note: CI, confidence interval; ROR, reporting odds ratio; N, number of drug-event pairs with the specific PT where isotretinoin was used concomitantly with the listed medication; PT, preferred term.

Among isotretinoin users reporting musculoskeletal and connective tissue disorder AEs, the 20 most frequent concomitant medications are shown in Table 4. These medications belonged to multiple classes, including oral contraceptives, antibiotics, antihistamines, antidepressants, analgesics, and a glucocorticoid (prednisone). Considering their high co-reporting frequency and reported association musculoskeletal AEs, we performed stratified ROR analyses for oral contraceptives, tetracyclines, and prednisone to characterize the reporting patterns of isotretinoin-related musculoskeletal and connective tissue disorder AEs during concomitant use (Figure 6). Overall, all three medication groups exhibited positive disproportionate reporting signals for common manifestations such as arthralgia and myalgia. Osteopenia demonstrated consistently high positive signals across all three drug classes, whereas estimates for PTs were based on small report counts and warrant cautious interpretation.

**TABLE 3:** The five most common preferred terms (PTs) for musculoskeletal and connective tissue disorder adverse events across isotretinoin dose subgroups.

Isotretinoin dose	PT	Number of reports	Proportion (%)
<0.5	Arthralgia	97	48.7%
​	Myalgia	51	25.6%
​	Back pain	42	21.1%
​	Arthritis	11	5.5%
​	Muscle spasms	8	4.0%
0.5≤and≤1	Arthralgia	160	44.3%
​	Back pain	113	31.1%
​	Myalgia	73	20.2%
​	Pain in extremity	17	4.7%
​	Neck pain	13	3.6%
>1	Arthralgia	47	44.3%
​	Myalgia	19	17.9%
​	Back pain	16	15.1%
​	Muscular weakness	5	4.7%
​	Bone pain	4	3.8%

Isotretinoin dose units are mg/kg/day. PT, preferred term.

**TABLE 4:** The 20 most frequently reported concomitant medications for adverse events of musculoskeletal and connective tissue disorders associated with isotretinoin.

Concomitant medications	Number of reports
Norgestimate and ethinyl estradiol	52
Acetaminophen	35
Cetirizine hydrochloride	32
Fexofenadine hydrochloride	30
Prednisone	28
Ibuprofen	27
Sertraline hydrochloride	27
Vitamin D	26
Multivitamin	25
Levothyroxine sodium	24
Oral contraceptive	23
Doxycycline hyclate	22
Cephalexin	22
Erythromycin	22
Fish oil	21
Escitalopram oxalate	21
Loratadine	21
Alprazolam	21
Tetracycline	20
Albuterol salbutamol	20

Drug name normalization was performed using the DailyMed (https://dailymed.nlm.nih.gov).

### 3.4 Time-to-onset analysis

**FIGURE 7:**
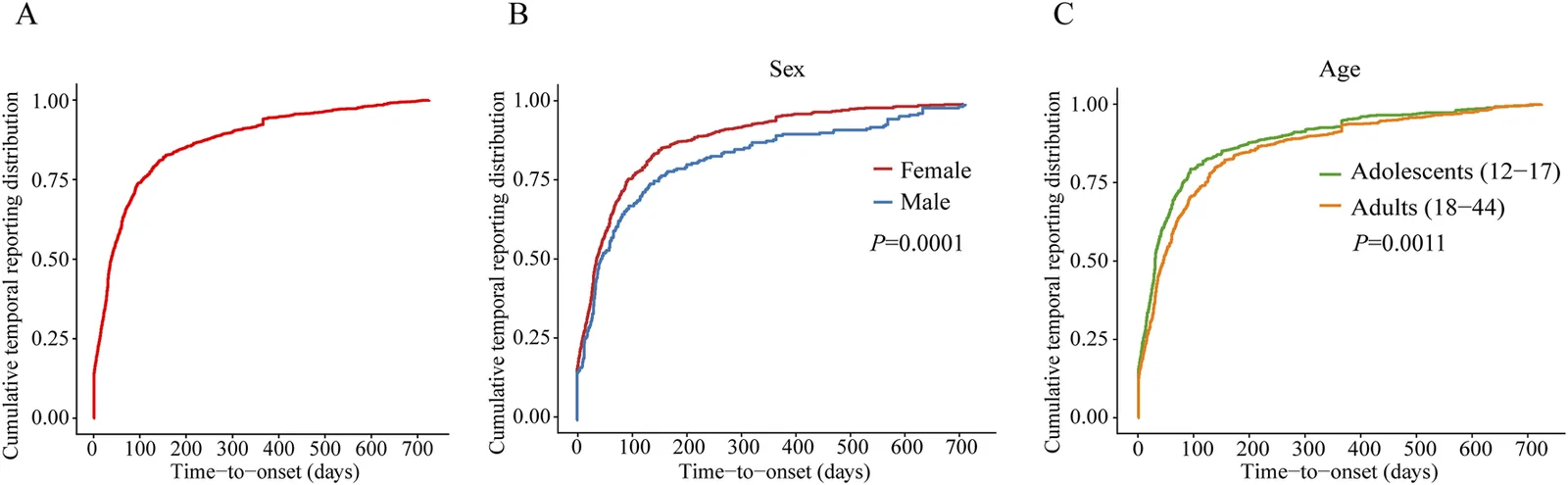
Cumulative temporal reporting distribution curves of musculoskeletal and connective tissue disorder adverse events among isotretinoin users **(A)** Overall population **(B)** Stratified by sex (female and male) **(C)** Stratified by age (adolescents and adults).

The time-to-onset of isotretinoin-related musculoskeletal and connective tissue disorder AEs was extracted from the FAERS database. After excluding reports with missing or implausible dates, 1,675 A E reports included valid time-to-onset data. The median time-to-onset was 38.0 days (interquartile range (IQR) 14.0–106.5 days). Weibull distribution analysis revealed a shape parameter (*β*) of 0.69 (95% *CI*: 0.66–0.72), indicating an early failure type pattern, where the majority of events occurred shortly after isotretinoin initiation. Consistent with the overall cohort, all subgroups exhibited an early failure type pattern (Table 5). Cumulative temporal reporting distribution curves were constructed to visualize event onset patterns across subgroup (Figure 7). Subgroup comparisons utilizing these curves revealed significant differences in the timing of event reporting. Specifically, females exhibited a significantly more rapid accumulation of reports compared to males (*P* = 0.0001). Furthermore, a significantly shorter time-to-onset trajectory was observed in adolescents (aged 12–17 years) compared to adults (aged 18–44 years) (*P* = 0.0011), indicating that younger populations tend to report these AEs earlier in their treatment course.

**TABLE 5:** Time-to-onset analysis for musculoskeletal and connective tissue disorder adverse events among isotretinoin users in the overall population and different strata.

Categories	N	Time-to-onset (days)	Weibull distribution analysis		Failure type
		Median (IQR)	Scale parameter: α (95% CI)	Shape parameter: β (95% CI)	​
Overall	1,675	38.0 (14.0–106.5)	69.26 (64.28, 76.07)	0.69 (0.66, 0.72)	Early
Female	847	36.0 (10.0–91.0)	61.68 (54.40, 69.33)	0.68 (0.65, 0.71)	Early
Male	229	43.0 (15.0–142.0)	94.60 (74.68, 119.13)	0.74 (0.65, 0.83)	Early
Adolescents	629	31.0 (10.0–81.0)	58.42 (50.88, 66.99)	0.68 (0.64, 0.72)	Early
Adults	809	46.0 (16.0–122.0)	74.48 (64.94, 82.62)	0.69 (0.66, 0.73)	Early

CI, confidence interval; N, number of reports with available time-to-onset.

### 3.5 Supplementary analysis

**FIGURE 8:**
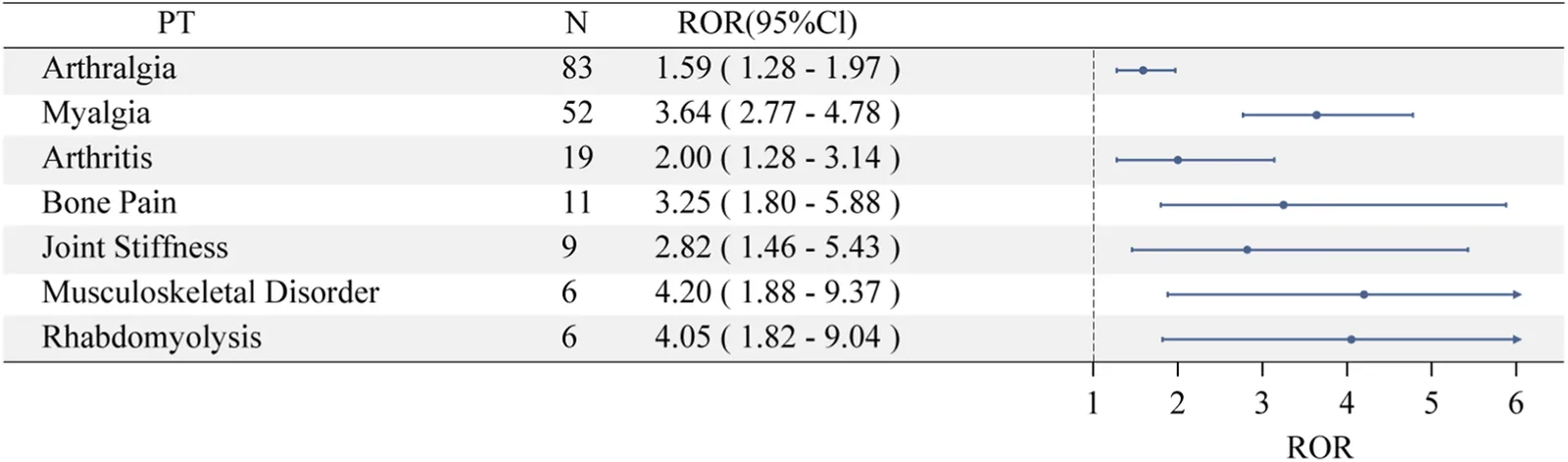
Reporting odds ratios (ROR) with 95% confidence intervals (CI) for positive signals of adverse events of musculoskeletal and connective tissue disorders among isotretinoin users in CVARD database. Note: CI, confidence interval; ROR, reporting odds ratio; N, number of drug-event pairs with suspected adverse events; PT, preferred term, CVARD, Canada Vigilance Adverse Reaction Online Database.

To further enrich the analyses of isotretinoin-related musculoskeletal AEs, we performed supplementary analyses using data from the CVARD database and clinical case series. As shown in Figure 8, the CVARD analysis identified seven safety signals. The most frequently reported signals were arthralgia (*N* = 83; ROR = 1.59) and myalgia (*N* = 52; ROR = 3.64), followed by arthritis (*N* = 19; ROR = 2.00), bone pain (*N* = 11; ROR = 3.25), joint stiffness (*N* = 9; ROR = 2.82), musculoskeletal disorder (*N* = 6; ROR = 4.20), and rhabdomyolysis (*N* = 6; ROR = 4.05). Notably, arthralgia, arthritis, and myalgia were consistently positive across both CVARD and FAERS, whereas bone pain, joint stiffness, musculoskeletal disorder, and rhabdomyolysis were not identified as positive signals in FAERS.

Clinical data were collected from patients who received isotretinoin at the Affiliated Hospital of Qingdao University between 1 January 2025, and 31 December 2025. A total of 25 cases were identified, comprising 10 males and 15 females, with a median age of 25 years (IQR: 20–28). The median time-to-onset was 66 days (IQR: 41–91), notably longer than the 38 days observed in FAERS, which may reflect differences in reporting practices or limited sample size. Back pain was the most frequently observed PT (*N* = 10), followed by Arthralgia (*N* = 2), Shoulder Pain (*N* = 2), Ganglion Cyst (*N* = 2), Muscle Twitching (*N* = 2), and Sacroiliitis (*N* = 2) (Table 6). These findings provide supplementary external contextualization for the FAERS-derived signals for isotretinoin-related musculoskeletal AEs.

**TABLE 6:** Frequency of musculoskeletal and connective tissue disorder preferred terms (PTs) in the single-center hospital clinical case series (*N* = 25).

PT	Number of cases
Back pain	10
Arthralgia	2
Shoulder pain	2
Ganglion cyst	2
Muscle twitching	2
Sacroiliitis	2
Neck pain	1
Myofascial pain syndrome	1
Musculoskeletal chest pain	1
Intervertebral disc protrusion	1
Costochondritis	1

PT, preferred term; each patient contributed only one PT, to this table.

## 4 Discussion

Isotretinoin, a retinoid and vitamin A derivative, exerts its anti-acne effects through activation of the RAR/RXR signaling pathway (Berbis, 2010). The RAR signaling pathway is essential for skeletal growth, extracellular matrix homeostasis, and growth plate function (Williams et al., 2009). Isotretinoin may disrupt cartilage and connective tissue homeostasis, thereby predisposing to musculoskeletal AEs.

This study identified 5,574 reports of isotretinoin-related musculoskeletal and connective tissue disorder AEs from the FAERS database, comprising 2,796 females (50.2%) and 2,257 males (40.5%). Our stratified disproportionality analysis at the SOC level was performed for isotretinoin associated musculoskeletal and connective tissue AEs. Males and adolescents exhibited significantly higher reporting RORs. Furthermore, among evaluated clinical variables, acne-related indications yielded the highest ROR, consistent with the primary therapeutic indication of the drug. Isotretinoin showed disproportionate reporting signals for musculoskeletal AEs in the FAERS database, whereas the topical retinoids did not show such signals in their respective database-wide analyses. These findings are consistent with a previous pharmacovigilance study by Szilagyi et al. and suggest that musculoskeletal AEs are primarily a systemic consequence of oral retinoid exposure, rather than a general class effect shared with localized topical therapies. Stratification by reporter type at the SOC level showed positive RORs across all subgroups, with the highest estimate observed among healthcare professionals. However, because a PT-level sensitivity analysis excluding lawyer-submitted reports was not performed, this SOC-level stratification cannot exclude the possibility that event-specific notoriety or litigation-related reporting bias contributed to individual PT signals, particularly for the emerging signals.

The most frequently reported AEs were arthralgia, back pain, and myalgia, consistent with previous clinical studies (Karaosmanoğl et al., 2020; Acar et al., 2022). To distinguish potentially emerging safety signals from established adverse drug reactions, we systematically compared the 30 FAERS-derived positive signals with current official isotretinoin product information across three regulatory jurisdictions: China (NMPA), the United States (FDA), and Canada (Health Canada) (Supplementary Table S5). Our analysis identified that 11 of the identified signals are already well-documented in the current label, including common events like arthralgia, back pain, and myalgia, as well as specific skeletal issues such as epiphyses premature fusion, bone hypertrophy, and ligament/tendon calcification. Furthermore, sacroiliitis is currently listed exclusively in the Canadian label, while osteopenia is noted in both the US and Canadian labels as a spontaneously reported event without established causality. Notably, our pharmacovigilance analysis identified 19 disproportionate reporting signals that are not currently described in the reviewed product labels. These unlabeled events include growth retardation, intervertebral disc degeneration, SAPHO syndrome, and costochondritis, among others. They represent potentially emerging safety signals associated with isotretinoin use and suggest the need for further investigation. The occurrence of these AEs may partly reflect effects of retinoid signalling on bone and joint tissues, as reported in previous mechanistic studies. Retinoic acid inhibits chondrocyte proliferation and cartilage matrix synthesis (Alazawi and Hendriksz, 2021; De Luca et al., 2000; Morales and Roberts, 1992). Isotretinoin may suppress osteoblast differentiation and enhance osteoclast activity, thereby disrupting the balance between bone resorption and formation (Kindmark et al., 1998; Ferreira et al., 2026). Additionally, isotretinoin upregulates matrix metalloproteinase (MMP) expression and alters the levels of proinflammatory cytokines, contributing to joint inflammation (Reyes-Hadsall et al., 2024; Malemud, 2017).

Notably, this study detected a disproportionate reporting signal for SAPHO syndrome. SAPHO (synovitis, acne, pustulosis, hyperostosis, and osteitis) syndrome is a rare autoinflammatory disorder characterized by cutaneous and osteoarticular manifestations (Demirci Yildirim and Sari, 2024). Research on isotretinoin-associated SAPHO syndrome has been limited to isolated case reports (Luzzati et al., 2020; Galadari et al., 2009), precluding establishment of a causal relationship. Despite its rarity, the exceptionally high signal intensity (ROR = 76.41) underscores the importance of considering SAPHO syndrome in the differential diagnosis for patients with refractory acne who develop persistent osteoarticular symptoms during isotretinoin therapy. Acne itself represents one clinical component of SAPHO syndrome. Given the small number of SAPHO-related reports, this study cannot establish a causal relationship between isotretinoin exposure and SAPHO syndrome; this study only detected a disproportionate reporting signal for SAPHO syndrome.

Sex-stratified analysis revealed higher RORs in males for arthralgia, arthritis, sacroiliitis, epiphyses premature fusion, and SAPHO syndrome. One possible hypothesis is that androgen-mediated regulation of immune and inflammatory pathways may contribute to this imbalance, as animal studies have demonstrated that androgen receptor signaling regulates Th17 cell metabolism and function, thereby influencing interleukin-17 expression (Mani and Weinberg, 2024), and that the interleukin-17/interleukin-23 axis drives spondyloarthritis pathogenesis (Srinath et al., 2024). Additionally, males experience a later and more prolonged pubertal growth spurt, with growth plates in a more active proliferative state during typical treatment ages; this may represent a potential mechanism underlying the higher RORs observed for epiphyses premature fusion (McGuire and Lawson, 1987; Hansen and Kjaer, 2016). However, these mechanistic interpretations remain hypothesis-generating and require further validation in independent studies with adequate control for confounding factors. In the unadjusted analysis, males showed a numerically lower ROR ratio for osteopenia than females; however, this difference was not statistically significant after Benjamini–Hochberg FDR correction. Mechanistically, estrogen has been shown to directly inhibit RANKL-stimulated osteoclast differentiation in human monocytes via ERα-BCAR1/TRAF6 interaction (Robinson et al., 2009). Therefore, this finding should be regarded as an exploratory observation. Whether this trend represents a true sex-specific effect of isotretinoin or underlying baseline differences in bone mineral density requires further investigation with objective bone density measurements. Sex-stratified cumulative temporal reporting distribution curves indicated earlier AE reporting among females compared with males. Weibull-distribution analysis demonstrated that both female and male groups belonged to the early-failure pattern, reflecting that musculoskeletal AEs predominantly arose in the early phase of isotretinoin exposure across both sexes.

Adolescents exhibited higher signal intensities for common musculoskeletal symptoms, including arthralgia and back pain, compared with adults, consistent with characteristics of rapid skeletal growth, increased physical activity, and heightened pain sensitivity in this population (Kujala et al., 1999). Notably, signal intensities for tendonitis, costochondritis, and intervertebral disc degeneration were markedly elevated in adolescents relative to adults, whereas epiphyses premature fusion was predominantly reported in adolescents (n = 66 vs. 2 adults) with a non-estimable subgroup comparison. *In vivo*, isotretinoin is isomerized to all-trans retinoic acid, which may accelerate growth plate closure through upregulation of ITGB2, activation of YAP, and inhibition of the Wnt/β-catenin signaling pathway (Xuan et al., 2025). Additionally, retinoic acid-induced protein 14 (RAI14) responds to mechanical forces and regulates Hippo signaling through interaction with NF2 (Jeong et al., 2024). Animal studies have demonstrated that isotretinoin induces Achilles tendinopathy in rats and adversely affects tendon biomechanical properties and histopathological structure (Beytemür et al., 2018). Correlation analysis revealed a weak but statistically significant association between epiphyses premature fusion and knee deformity (*r* = 0.255, *P* < 0.001). Collectively, these disproportionate reporting signals and pre-clinical mechanistic findings suggest that heightened clinical vigilance for activity related pain at weight bearing sites may be reasonable for adolescent patients receiving isotretinoin. Age-stratified cumulative temporal reporting distribution curves revealed earlier AE reporting among adolescents relative to adults. Weibull-distribution analysis showed that both adolescents and adults exhibited an early failure pattern, indicating that musculoskeletal AEs predominantly occurred within the early phase of isotretinoin exposure in both age subgroups.

Dose-stratified analyses were performed to characterize musculoskeletal AEs across different isotretinoin dose groups, and concomitant medication analyses were conducted to explore pattern variations when isotretinoin was co-administered with other agents. Arthralgia and myalgia were the most frequently reported events across all dose subgroups, with no significant differences among dose groups. In contrast, a statistically significant difference was observed for back pain, with the highest proportion in the 0.5–1 mg/kg/day group (31.1%). However, given that dose information was missing for the vast majority of reports, and multiple outcomes were examined, this finding should be interpreted cautiously as exploratory rather than as evidence of a dose-response relationship. Previous studies on dose-effect relationships have been inconsistent: Karaosmanoğl et al., 2020. reported a positive correlation between isotretinoin-induced lower back pain and cumulative dose (Karaosmanoğl et al., 2020), whereas Acar et al. found no association between cumulative dose and back pain incidence, but noted a positive correlation with pain severity (Acar et al., 2022). Under different concomitant medication scenarios, arthralgia and myalgia remained predominant, although distinct pattern variations could not be reliably assessed due to small report numbers. Tetracyclines have been previously linked to musculoskeletal events (Warner et al., 2022), and prednisone has been associated with potential musculoskeletal events (Hartmann et al., 2016). Overall, further well controlled studies accounting for confounding factors and indication bias are needed to clarify the effects of dose and concomitant medications on musculoskeletal AEs.

We supplemented our FAERS analysis with CVARD data and a single-center clinical case series. Three signals (arthralgia, arthritis, and myalgia) were consistently detected in both FAERS and CVARD. Several of these signals, including back pain, arthralgia, shoulder pain, sacroiliitis, and costochondritis, were also observed in the single-center clinical case series. However, the concordance across datasets was limited, likely due to differences in population composition, data sources, and sample size. Therefore, larger-scale sensitivity analyses are needed to confirm these FAERS preliminary signals.

### 4.1 Limitations

This study provides a comprehensive pharmacovigilance profile of isotretinoin-associated musculoskeletal AEs; however, several limitations should be noted. First, FAERS is a spontaneous reporting database that lacks total exposure denominators, precluding true incidence rate estimation. The data are inherently susceptible to underreporting, stimulated reporting, and litigation related bias, including attorney-submitted reports. Furthermore, the single-center clinical case series lacked a comparator group and had a modest sample size, limiting external generalizability. Second, substantial missing information, including drug dosage, time-to-onset, and basic demographic variables such as age (35.8% missing) and sex (9.3% missing), required patient exclusions, which may have introduced selection bias and reduced generalizability. Third, disproportionality analysis reflects statistical associations rather than definite causality. Findings remain vulnerable to confounding by indication, disease severity, and potential reverse causation, including protopathic bias. Moreover, the concomitant medication analysis is limited by the inability to establish temporal sequencing and differential reporting, as these agents may have been initiated after AE onset or used to treat the event, thereby introducing indication bias and protopathic bias. Finally, analysis of rare events with small event counts warrants caution, as sparse data may result in wide confidence intervals and imprecise effect estimates. These findings should be viewed as hypothesis generating and require verification in prospective, multicenter cohorts.

## 5 Conclusion

In conclusion, this study detected several potential disproportionality signals associated with isotretinoin, including SAPHO syndrome, intervertebral disc degeneration, and costochondritis, among others, that appear to be insufficiently documented in current product labeling. Sex- and age-stratified analyses identified several exploratory differences in reporting patterns. Male-predominant signals were observed for SAPHO syndrome, epiphyses premature fusion, sacroiliitis, arthritis, and arthralgia. Adolescents demonstrated higher reporting RORs than adults for arthralgia, back pain, tendonitis, intervertebral disc degeneration, and costochondritis. Epiphyses premature fusion was predominantly reported in adolescents (n = 66, vs 2 adults), but the ROR for the adolescent versus adult comparison was not estimable owing to sparse data. Importantly, these subgroup findings were not adjusted for other potential confounding factors and should therefore be considered exploratory and hypothesis-generating. Further validation using appropriately adjusted and prospective studies is warranted.

## Data Availability

The original contributions presented in the study are included in the article/Supplementary Material, further inquiries can be directed to the corresponding author.
