# Supplementary material for: Sex- and age-specific musculoskeletal adverse events of isotretinoin: disproportionality analysis of the FAERS database combined with a clinical case series
Source: Front Pharmacol. 2026 Sep 11;17:1904582. doi: 10.3389/fphar.2026.1904582 (PMC13612492; doi:10.3389/fphar.2026.1904582)
Supplement: Supplementary file 1 [file Supplementaryfile1.docx]

**Supplementary Figure S1. Annual number of reports of isotretinoin-related adverse events submitted to the FAERS from 2004 to 2025.**

**Supplementary Figure S2. Flowchart of patient screening for isotretinoin-related musculoskeletal adverse events in the single-center clinical case series**

**Supplementary Table S1. Baseline characteristics of the overall cohort and reports with known sex.**

| Characteristic | Overall (n=5574) | Sex-known (n=5053) | Sex-missing (n=521) | *P*-value |
| --- | --- | --- | --- | --- |
| **Age** |  |  |  | <0.001 |
| 0-11 | 54(1.0%) | 53 (1.0%) | 1 (0.2%) |  |
| 12-17 | 1358(24.3%) | 1,291(25.7%) | 67 (12.9%) |  |
| 18-44 | 1916(34.4%) | 1,866 (37%) | 50 (9.6%) |  |
| 45-59 | 208(3.7%) | 198 (3.9%) | 10 (1.9%) |  |
| >59 | 45(0.8%) | 40 (0.8%) | 5 (1.0%) |  |
| Missing | 1993(35.8%) | 1,605 (32%) | 388 (74.5%) |  |
| **Weight(kg)** |  |  |  | <0.001 |
| ＜50 | 193(3.4%) | 191 (3.8%) | 2 (0.4%) |  |
| 50≤and≤100 | 1967(35.3%) | 1957(39%) | 10 (1.9%) |  |
| ＞100 | 131(2.4%) | 131(2.6%) | 0 (0%) |  |
| Missing | 3283(58.9%) | 2774(55%) | 509 (97.7%) |  |
| **Reporters** |  |  |  | <0.001 |
| Physician (MD) | 1730(31.0%) | 1,452 (29%) | 278 (53.4%) |  |
| Consumer (CN) | 1487(26.7%) | 1,360 (27%) | 127 (24.4%) |  |
| Lawyer (LW) | 1105(19.8%) | 1,102 (22%) | 3 (0.6%) |  |
| Other health-professional (OT) | 625(11.2%) | 573 (11%) | 50 (9.6%) |  |
| Health professional (HP) | 372(6.7%) | 319 (6.3%) | 53 (10.2%) |  |
| Pharmacist (PH) | 64(1.1%) | 59 (1.2%) | 5 (1.0%) |  |
| Missing | 191(3.4%) | 188(3.7%) | 5 (1.0%) |  |
| **Reported country**  **(Top three)** |  |  |  | <0.001 |
| United States of America (US) | 4503(80.8%) | 4,047 (80%) | 456 (87.5%) |  |
| United Kingdom of Great Britain and Northern Ireland (GB) | 134(2.4%) | 106 (2.1%) | 28 (5.4%) |  |
| The Republic of Turkey (TR) | 124(2.3%) | 116 (2.3%) | 10 (1.9%) |  |

Note: *P*-values were calculated using the χ² test for comparisons sex-known and sex-missing reports.

**Supplementary Table S2. Baseline characteristics stratified by age group: overall cohort, 12-17 years and 18-44 years**

| Characteristic | All (n=5574) | Adolescents (n = 1358) | Adults  (n = 1916) | P-value |
| --- | --- | --- | --- | --- |
| **Sex** |  |  |  | <0.001 |
| Female | 2796 (50.2%) | 443 (32.6%) | 1187 (62.0%) |  |
| Male | 2257 (40.5%) | 848 (62.4%) | 679 (35.4%) |  |
| Missing | 521 (9.3%) | 67 (4.9%) | 50 (2.6%) |  |
| **Weight (kg)** |  |  |  | 0.133 |
| <50 | 193 (3.5%) | 46 (3.4%) | 70 (3.7%) |  |
| 50≤and≤100 | 1967 (35.5%) | 610 (44.9%) | 870 (45.4%) |  |
| >100 | 131 (2.4%) | 25 (1.8%) | 59 (3.1%) |  |
| Missing | 3283 (58.9%) | 677 (49.9%) | 917 (47.9%) |  |
| **Reporters** |  |  |  | <0.001 |
| Physician (MD) | 1730 (31.0%) | 429 (31.6%) | 531 (27.7%) |  |
| Consumer (CN) | 1487 (26.7%) | 304 (22.4%) | 362 (18.9%) |  |
| Lawyer (LW) | 1105 (19.8%) | 284 (20.9%) | 554 (28.9%) |  |
| Other health-professional (OT) | 625 (11.2%) | 162 (11.9%) | 236 (12.3%) |  |
| Health professional (HP) | 372 (6.7%) | 100 (7.4%) | 121 (6.3%) |  |
| Pharmacist (PH) | 64 (1.1%) | 19 (1.4%) | 26 (1.4%) |  |
| Missing | 191 (3.4%) | 60 (4.4%) | 86 (4.5%) |  |
| **Reporter country (Top three)** |  |  |  | <0.001 |
| United States of America (US) | 4503 (80.8%) | 1033 (76.1%) | 1450 (75.7%) |  |
| The Republic of Turkey (TR) | 126 (2.3%) | 20 (1.5%) | 81 (4.2%) |  |
| United Kingdom of Great Britain and Northern Ireland (GB) | 134 (2.4%) | 39 (2.9%) | 42 (2.2%) |  |

Note: *P*-values were calculated using the χ² test for comparisons between the adolescent and adult groups.

**Supplementary Table S3. Stratified ROR ratios for isotretinoin-related musculoskeletal adverse events by sex and age group.**

| PT | Male (n) | Female (n) | Male/Female  ROR Ratio (95% CI) | Adolescent (n) | Adult (n) | Adolescent/Adult ROR Ratio (95% CI) |
| --- | --- | --- | --- | --- | --- | --- |
| Arthralgia | 926 | 1228 | 1.42 (1.30-1.54)* | 564 | 801 | 2.30 (2.02-2.62)* |
| Back Pain | 436 | 618 | 1.16 (1.02-1.31) | 330 | 372 | 3.30 (2.83-3.85)* |
| Myalgia | 398 | 530 | 1.03 (0.91-1.19) | 398 | 331 | 1.07 (0.92-1.24) |
| Arthritis | 136 | 162 | 1.77 (1.37-2.29)* | 48 | 160 | 0.85 (0.61-1.19) |
| Sacroiliitis | 97 | 75 | 1.89 (1.41-2.53)* | 49 | 107 | 0.98 (0.61-1.55) |
| Osteopenia | 50 | 86 | 0.70 (0.50-0.97) | 28 | 76 | 1.44 (0.89-2.33) |
| Epiphyses Premature Fusion | 74 | 22 | 2.72 (1.47-5.04)* | 66 | 2 | NA |
| Fistula | 53 | 36 | 1.50 (1.00-2.25) | 23 | 43 | 1.35 (0.80-2.29) |
| Tendonitis | 39 | 37 | 1.55 (0.99-2.42) | 24 | 24 | 7.41 (4.08-13.45)* |
| Growth Retardation | 36 | 8 | 2.08 (0.97-4.44) | 34 | 1 | NA |
| Intervertebral Disc Degeneration | 19 | 23 | 1.45 (0.78-2.68) | 8 | 26 | 5.57 (2.05-15.13)* |
| SAPHO Syndrome | 29 | 5 | 7.85 (2.89-21.33)* | 25 | 6 | 1.97 (0.71-5.44) |
| Costochondritis | 5 | 11 | 1.39 (0.48-4.02) | 9 | 6 | 5.44 (1.83-16.18)* |
| Arthritis Enteropathic | 5 | 11 | 0.84 (0.28-2.51) | 3 | 11 | 1.12 (0.22-5.66) |
| Knee Deformity | 8 | 4 | 2.96 (0.89-9.89) | 2 | NA | NA |
| Growth Failure | 7 | 4 | 1.36 (0.39-4.75) | 7 | NA | NA |
| Torticollis | 5 | 4 | 1.34 (0.36-5.02) | 3 | 1 | NA |
| Joint Noise | 2 | 7 | NA | 2 | 6 | NA |
| Osteochondrosis | 6 | 2 | NA | 3 | 2 | NA |
| Bone Hypertrophy | 5 | 3 | 2.06 (0.47-9.05) | NA | 8 | NA |
| Enthesopathy | 2 | 6 | NA | NA | 5 | NA |
| Patellofemoral Pain Syndrome | 3 | 4 | 1.67 (0.37-7.59) | 4 | 2 | NA |
| Growth Disorder | 4 | NA | NA | 5 | NA | NA |
| Head Deformity | 5 | 2 | NA | NA | 1 | NA |
| Shoulder Pain | 4 | 2 | NA | 2 | 4 | NA |
| Arthritis Reactive | 3 | 3 | 0.71 (0.14-3.57) | NA | 5 | NA |
| Ligament Calcification | 4 | 1 | NA | 4 | 1 | NA |
| Tendon Calcification | 4 | 1 | NA | 4 | 1 | NA |
| Epiphyseal Disorder | 4 | 1 | NA | 3 | NA | NA |
| Spondyloarthropathy | 2 | 3 | NA | NA | 4 | NA |

Note: NA (not estimable), ROR ratios and 95% CIs were not calculated when either subgroup contained fewer than 3 reports; n, indicates the number of drug-event pairs for the specific adverse event within each subgroup; *, *P* <0.05 after Benjamini-Hochberg FDR correction.

**Supplementary Table S4. Comparison of event proportions for selected preferred terms across isotretinoin dose groups.**

| **PT** | <0.5 mg/kg/day  (*N*=199) | 0.5≤and≤1mg/kg/day (*N*=361) | >1 mg/kg/day  (*N*=106) | *P*-value |
| --- | --- | --- | --- | --- |
| Arthralgia | 97(48.7%) | 160(44.3%) | 47(44.3%) | 0.578 |
| Myalgia | 51(25.6%) | 73(20.2%) | 19(17.9%) | 0.206 |
| Back Pain | 42(21.1%) | 113(31.1%) | 16(15.1%) | 0.001 |

*P*-values were calculated using *χ*^2^ test.

**Supplementary Table S5. Comparison of FAERS-derived disproportionate reporting signals with isotretinoin product-labeled adverse reactions.**

| **PT** | **isotretinoin product information** |
| --- | --- |
| Arthralgia | Isotretinoin soft capsules, 10 mg (China, NMPA approval No. H20234744) ABSORICA™ (isotretinoin) capsules, oral capsules (initial U.S. FDA approval)  ACCUTANE™ (isotretinoin) capsules, 10 mg, 40 mg (Canada, Health Canada, DIN 00582344) |
| Back Pain | Isotretinoin soft capsules, 10 mg (China, NMPA approval No. H20234744) ABSORICA™ (isotretinoin) capsules, oral capsules (initial U.S. FDA approval)  ACCUTANE™ (isotretinoin) capsules, 10 mg, 40 mg (Canada, Health Canada, DIN 00582344) |
| Myalgia | Isotretinoin soft capsules, 10 mg (China, NMPA approval No. H20234744) ABSORICA™ (isotretinoin) capsules, oral capsules (initial U.S. FDA approval)  ACCUTANE™ (isotretinoin) capsules, 10 mg, 40 mg (Canada, Health Canada, DIN 00582344) |
| Arthritis | Isotretinoin soft capsules, 10 mg (China, NMPA approval No. H20234744) ABSORICA™ (isotretinoin) capsules, oral capsules (initial U.S. FDA approval)  ACCUTANE™ (isotretinoin) capsules, 10 mg, 40 mg (Canada, Health Canada, DIN 00582344) |
| Sacroiliitis | ACCUTANE™ (isotretinoin) capsules, 10 mg, 40 mg (Canada, Health Canada, DIN 00582344) |
| Osteopenia | ABSORICA™ (isotretinoin) capsules, oral capsules (initial U.S. FDA approval)*  ACCUTANE™ (isotretinoin) capsules, 10 mg, 40 mg (Canada, Health Canada, DIN 00582344)* |
| Epiphyses Premature Fusion | Isotretinoin soft capsules, 10 mg (China, NMPA approval No. H20234744) ABSORICA™ (isotretinoin) capsules, oral capsules (initial U.S. FDA approval)  ACCUTANE™ (isotretinoin) capsules, 10 mg, 40 mg (Canada, Health Canada, DIN 00582344) |
| Fistula | NA |
| Tendonitis | Isotretinoin soft capsules, 10 mg (China, NMPA approval No. H20234744) ABSORICA™ (isotretinoin) capsules, oral capsules (initial U.S. FDA approval)  ACCUTANE™ (isotretinoin) capsules, 10 mg, 40 mg (Canada, Health Canada, DIN 00582344) |
| Growth Retardation | NA |
| Intervertebral Disc Degeneration | NA |
| SAPHO Syndrome | NA |
| Costochondritis | NA |
| Arthritis Enteropathic | NA |
| Knee Deformity | NA |
| Growth Failure | NA |
| Torticollis | NA |
| Joint Noise | NA |
| Osteochondrosis | NA |
| Bone Hypertrophy | Isotretinoin Soft Capsules（China, NMPA approval No. H20234744） ABSORICA™ (isotretinoin) capsules, oral capsules (initial U.S. FDA approval)  ACCUTANE™ (isotretinoin) capsules, 10 mg, 40 mg (Canada, Health Canada, DIN 00582344) |
| Enthesopathy | NA |
| Patellofemoral Pain Syndrome | NA |
| Growth Disorder | NA |
| Head Deformity | NA |
| Shoulder Pain | NA |
| Arthritis Reactive | NA |
| Ligament Calcification | Isotretinoin soft capsules, 10 mg (China, NMPA approval No. H20234744) ABSORICA™ (isotretinoin) capsules, oral capsules (initial U.S. FDA approval)  ACCUTANE™ (isotretinoin) capsules, 10 mg, 40 mg (Canada, Health Canada, DIN 00582344) |
| Tendon Calcification | Isotretinoin soft capsules, 10 mg (China, NMPA approval No. H20234744) ABSORICA™ (isotretinoin) capsules, oral capsules (initial U.S. FDA approval)  ACCUTANE™ (isotretinoin) capsules, 10 mg, 40 mg (Canada, Health Canada, DIN 00582344) |
| Epiphyseal Disorder | NA |
| Spondyloarthropathy | NA |

Note: *, Spontaneous reports of osteopenia have been seen in the isotretinoin population, while causality with isotretinoin has not been established. NMPA, National Medical Products Administration; FDA, Food and Drug Administration; NA, not available, neither the exact MedDRA PT nor any related term at the HLT/HLGT level was identified in the product label after hierarchical concept mapping.
